# Efficacy and Safety of GHX02 in the Treatment of Acute Bronchitis and Acute Exacerbation of Chronic Bronchitis: A Phase Ⅱ, Randomized, Double-Blind, Placebo-Controlled, Multicenter Trial

**DOI:** 10.3389/fphar.2021.761575

**Published:** 2022-01-17

**Authors:** Su Won Lee, Yee Ran Lyu, Si Yeon Kim, Won Kyung Yang, Seung Hyung Kim, Ki Mo Kim, Sung-Wook Chae, Weechang Kang, In Chul Jung, Yang Chun Park

**Affiliations:** ^1^ Division of Respiratory Medicine, Department of Internal Medicine, College of Korean Medicine, Daejeon University, Daejeon, South Korea; ^2^ Korean Institute of Oriental Medicine, Daejeon, South Korea; ^3^ Department of Statistics, Hyehwa Liberal Arts College, Daejeon University, Daejeon, South Korea; ^4^ Institute of Traditional Medicine and Bioscience, Daejeon University, Daejeon, South Korea; ^5^ Department of Neuropsychiatry, College of Korean Medicine, Daejeon University, Daejeon, South Korea

**Keywords:** acute bronchitis, acute exacerbation of chronic bronchitis, Ghx02, herbal drug, Korean medicine

## Abstract

Acute bronchitis and acute exacerbations of chronic bronchitis (AECB) have cough and sputum as the main symptoms with a high prevalence and substantial economic burden. Although the demand for bronchitis treatment increases due to causes, such as air pollution, the appropriateness of antibiotic prescriptions and the effects of current symptomatic treatments for bronchitis are unclear. GHX02, which is a combined formulation containing four herbs, and has been clinically used for bronchitis in South Korea. We conducted a phase II, randomized, double-blind, and placebo-controlled, multicenter trial to evaluate its efficacy and safety. Patients with acute bronchitis or AECB were recruited and randomized to receive high-dose GHX02 (1920 mg/day), standard-dose GHX02 (960 mg/day), or placebo for 7 days. The primary outcome measure was the change in Bronchitis Severity Score (BSS) from baseline to Day 7. The secondary outcomes were the frequency of coughing fits, Questionnaire of Clinical Symptoms of Cough and Sputum (QCSCS), Leicester Cough Questionnaire (LCQ), Integrative Medicine Outcome Scale (IMOS), and Integrative Medicine Patient Satisfaction Scale (IMPSS). A total of 117 patients were randomized to parallel groups (38 in the high-dose GHX02, 41 in the standard-dose GHX02 group, and 38 in the placebo group). The mean differences in BSS from baseline to Day 7 in the treatment groups (4.2 ± 2.0 and 4.5 ± 1.8 in the high-dose GHX02 and standard-dose GHX02 groups, respectively) were higher than the placebo group (3.8 ± 2.1), *p* = 0.028. The mean differences in the frequency of coughing fits from baseline to Day 7 and IMPSS were better in the GHX02 treatment group than in the placebo group (standard-dose GHX02 group vs placebo group, *p* = 0.036). The QCSCS, LCQ, IMOS, and GHX02 of the treatment groups also showed more improvement than the placebo group, but there were no statistically significant differences between the groups. There were no severe adverse effects during the trial. This study supports that GHX02 is effective and safe for patients with bronchitis and provides the basis for progression to a phase III study.

**Clinical Trial Registration**: [https://cris.nih.go.kr] WHO International Clinical Trials Registry Platform, Clinical Research Information Service [KCT0003665].

## 1 Introduction

Acute bronchitis is defined as an acute lower respiratory tract infection disease characterized by cough with or without sputum, lasting no more than 3 weeks with no clinical or any recent radiographic evidence to suggest an alternative explanation (Smith et al., 2020). The diagnosis of acute bronchitis requires an assessment to differentiate from pneumonia, asthma as there are no specific diagnostic criteria, and a symptom scoring system is used clinically to evaluate severity. (Albert, 2010). Acute bronchitis is a common clinical disease responsible for both primary care clinicians and emergency department attendances (Woodhead et al., 2011). It is reported in up to 10% of the population, with approximately 100 million ambulatory visits per year in the United States (Grijalva et al., 2009). In South Korea, acute bronchitis is also the second most frequent outpatient department (OPD) disease, which is visited by 16 million patients and places a substantial economic burden on the public health system (Sun-min Kim, 2020). Acute exacerbation of chronic bronchitis (AECB) is another common disease with cough and sputum as the main symptoms. The overall rate of AECB episodes was reported to be up to 3% and increased between 1992 and 2000 (Feeney et al., 2004).

Acute bronchitis is mainly caused by a viral infection and less than 10% of bacterial infections; therefore, antibiotics do not have benefits in viral bronchitis (Braman, 2006). In addition, antibiotics have side effects on normal bacteria, resistance to respiratory pathogens, and cost of antibiotic treatment; however, the overall antibiotic prescription rate for acute bronchitis was 60–83% (Smith et al., 2017). In addition, in AECB, because 30% of AECB results from viruses and 20% of AECB are due to non-infectious causes, judicious use of antibiotics is recommended for differentiating between bacterial, and non-bacterial infections (Fendrick et al., 2001). Patients with cough/sputum for acute bronchitis and mild AECB are usually prescribed antitussives, expectorants, beta-2 agonists, and alternative therapies as symptomatic treatments. However, central antitussives have limited efficacy for bronchitis, and expectorants, such as mucolytic agents, and have no consistent favorable effects in several trials. As beta-2 agonist bronchodilators may be useful for wheezing accompanying cough, it should not be routinely used to alleviate cough due to its side effects (Braman, 2006). As alternative therapies, ivy leaf extracts are used in various European countries, and several trials have been conducted on Chinese medicinal herbs for acute bronchitis, but there is insufficient evidence to support its use (Holzinger and Chenot, 2010; Jiang et al., 2012).

In this study, we evaluated the efficacy and safety of a combined formulation GHX02, containing four herbs that have been clinically used for bronchitis at Daejeon Korean Medicine Hospital, Daejeon University. GHX02 originated from gwaruhaengryeon-hwan in Dong-Ui-Bo-Gam, the classical medical text approved by UNESCO in 2009. Gwaruhaengryeon-hwan is composed of three herbs (*Trichosanthis semen*, *Armeniacae semen,* and *Coptidis rhizoma*) known to stabilize lung-heat (肺熱), usually meaning inflammation and acute stage of the disease in syndrome differentiation of traditional oriental medicine. Here, we tried to expand the effect by adding a herb (*Scutellariae radix)* with a similar therapeutic mechanism. Gwaruhaengryeon-hwan demonstrated its anti-inflammatory effects on COPD and particulate matter-induced lung injury in a mouse model (Lee et al., 2017). *Scutellariae radix* has also been reported to inhibit the production of several inflammatory cytokines (Kim et al., 2015) and have antioxidant effects (Guo et al., 2013). Moreover, a series of preclinical studies showed that GHX02 reduced airway inflammation, sputum production, cough, suppressed PM10D-induced inflammatory symptoms in the lung (Yang et al., 2020), and had an anti-COPD effect (Yang et al., 2018). Additionally, the safety of GHX02 has been verified in the assessment of 4-weeks repeated-dose oral toxicity and genotoxicity (Ji et al., 2020). Although there are many animal studies and clinical experiences, there have been no clinical trials in humans that evaluate GHX02. Therefore, we planned a phase II, randomized, double-blind, placebo-controlled, multicenter trial to determine the efficacy and safety of GHX02 compared with placebo, and find a suitable dosage of GHX02 for the treatment of bronchitis. This study is expected to provide clinical data for the following phase III trials.

## 2 Materials and Methods

The study protocol was approved by the Institutional Review Board (IRB) at each center (IRB number: DJDSKH-17-DR-14 at Daejeon Korean Medicine Hospital, KOMCIRB 2018-10-007-001 at Kyung Hee University Korean Medicine Hospital, and 2018010 at Pusan National University Korean Medicine Hospital). In contrast to the previously published protocol (Lyu et al., 2018), this study included patients with AECB with symptoms similar to those with acute bronchitis but excluded those who required glucocorticoids or antibiotic treatment. Accordingly, stratified block randomization was not conducted for Korean pattern identification, but as acute bronchitis and AECB.

### 2.1 Design and Procedures

This study was a phase II, randomized, double-blind, placebo-controlled, multicenter, and dose-finding trial to investigate the efficacy and safety of two different doses of GHX02 compared to placebo. The participants were recruited from three university-affiliated hospitals, including Daejeon Korean Medicine Hospital, Daejeon University, Kyung Hee University Korean Medicine Hospital, and Pusan National University Korean Medicine Hospital. The patients were screened by tests, including chest X-ray, electrocardiogram (ECG), and laboratory tests to rule out other diseases. The enrolled patients who met the eligibility criteria were stratified into acute bronchitis or AECB and randomized to parallel groups in a ratio of 1:1:1 for the high-dose GHX02 group (1920 mg/day), standard-dose GHX02 group (960 mg/day), and control group (placebo). The patient took one of the medications three times within 30 min to 1 h after meals in a day for 7 days, with two visiting days (Days 0 and 7). The outcome measures were performed before the medication (Day 0, visit 2) and after the medication (Day 7, visit 3). Safety was evaluated in terms of adverse events (AEs), vital signs at each visit, and laboratory examinations (liver function test and routine blood and urine tests) before and after taking the medication.

### 2.2 Participants

The trial included patients (age, 19–75 years) with a Bronchitis Severity Score (BSS) ≥5 points on Day 0 due to acute bronchitis or with increased respiratory symptoms due to AECB for more than 2 days. Written consent was obtained from all patients to participate in this study. The symptoms began within 2 weeks before the study, and the diagnoses of acute bronchitis were based on the patients’ medical history, physical examination, and BSS, including cough, sputum, dyspnea, chest pain during coughing, and rales on auscultation. Primary exclusion criteria were treatment with antitussives or expectorants during the last 7 days before the study and the history or presence of confounding severe respiratory diseases that may affect the evaluation of the efficacy of clinical medicine (e.g., pneumonia, cystic fibrosis, lung cancer, and or active pulmonary tuberculosis). A detailed list of inclusion and exclusion criteria is provided in the protocol (Lyu et al., 2018).

### 2.3 Sample Size

This study aimed to evaluate the efficacy of GHX02 in patients with bronchitis. The estimation of the number of subjects was based on whether there was a difference in the change from baseline to post-dose BSS between the control and treatment groups. Based on a previous study, we assumed that the difference would be reported to be 2.3 (μ*c—*μ*t* = 2.3), and the standard deviation (SD, σ) of the changes in BSS was assumed to be 3.2 (Matthys and Heger, 2007). With the power to detect a difference of 0.8 and a two-sided significance level of 0.05, 31 patients were required for each group. Because the ratio of allocation of patients between the groups was 1:1:1, and the dropout rate was presumed to be 0.2; therefore, this study recruited 39 participants to each group, totaling 117 participants.

### 2.4 Randomization and Blinding

Randomization was conducted using a computer random number generator in SAS Analytics Pro (SAS Institute) by an independent statistician. Allocation was implemented by the manufacturers who collectively labeled the participant’s identification codes with the packages of the test drug or placebo using generated random numbers. The management pharmacist gave the participant a labeled drug corresponding to the participant’s identification code. Only the statistician and manufacturer had access to the random numbers, and only the identification code was used to identify which drug to give to which participant. This was a double-blind trial; thus, neither participants nor investigators (including outcome assessors) were aware of group assignment until the end of the study period. Placebo tablets were matched to GHX02 tablets in terms of color, taste, smell, and outer packaging.

### 2.5 Interventions

GHX02 is a combination of four herbs: gwaruin (*Trichosanthis semen*, 351 mg), Haengin (*Armeniacae semen*, 175.5 mg), Hwangryeon (*Coptidis rhizoma*, 175.5 mg), and Hwanggeum (*Scutellariae radix*, 351 mg) (Table 1). One tablet of GHX02 (500 mg) contains 160.0 mg of dry extract of the four herbs (obtained by boiling in water and then dehydrating) mixed with 340.0 mg of starch and lactose. The trial medications were prepared by Hankookshinyak Corporation (Nonsan, Korea) according to the Korean Good Manufacturing Practice guidelines. The manufacturer complied with the regulations on the safety of pharmaceuticals and appropriately managed the quality. The placebo tablet was manufactured by the same company and did not contain any active ingredients. Both tablets had the same color, shape, smell, and taste.

**TABLE 1:** Components of GHX02.

Herb	Latin name	Family name	Part of plant	Amount (mg)
Gwaruin	*Trichosanthis semen*	Cucurbitaceae	Seed	351
Haengin	*Armeniacae semen*	Rosaceae	Seed	175.5
Hwangryeon	*Coptidis rhizoma*	Ranunculaceae	Root stock	175.5
Hwanggeum	*Scutellariae radix*	Labiatae/Lamiaceae	Root	351

The dosage was determined according to the pharmacologically active dose in the expectorant-effective tests. All enrolled patients were prescribed four tablets of either clinical medicine or placebo and administered three times daily for 7 days. To improve compliance, participants were asked to record their daily dose in the cough diary. The overall medication compliance during the trial should be at least 75%; if the medication compliance is less than 75%, the participant is considered an inadequate subject.

### 2.6 Outcomes

#### 2.6.1 Primary Outcome

The primary outcome of this study was a change in the BSS before and after the intervention (Day 0, Day 7). The BSS is an evaluation tool for acute bronchitis and is a valid clinical measure for initial diagnosis and treatment assessment (Lehrl et al., 2014). BSS is the sum of five major symptom scores: cough, sputum, dyspnea, chest pain during coughing, and rales on auscultation. Each symptom was scored on a 5-point scale (0 = absent, 1 = mild, 2 = moderate, 3 = severe, and 4 = very severe), with a maximum total score of 20 points**.** (Table 2). Because acute bronchitis mainly involves subjective complaints, the BSS score based on subjective symptoms is highly correlated with the actual improvement of the patient (Matthys and Kamin, 2013).

**TABLE 2:** Bronchitis severity score (BSS).

Assessment/Symptoms	Absent	Mild	Moderate	Severe	Very severe
Cough	0	1	2	3	4
Sputum	0	1	2	3	4
Dyspnea	0	1	2	3	4
Chest pain during coughing	0	1	2	3	4
Rales on auscultation	0	1	2	3	4

#### 2.6.2 Secondary Outcomes

For more reliable data, objective tools and subjective tools, and were included in the secondary outcomes. The frequency of coughing fits is an objective measurement tool to evaluate coughing. A cough diary was given to record their daily coughing frequency, and participants were classified on one of the following scales: 0 = 0 time/day, 1 = 1 time/day, 2 = 2–3 times/day, 3 = 4–5 times/day (sometimes), 4 = 6–10 times/day (frequent), and 5 = over 15 times/day (consistently) (Fischer and Dethlefsen, 2013). The Questionnaire of Clinical Symptoms of Cough and Sputum (QCSCS) was developed by modifying the Clinical Asthma Measurement Scale in Oriental Medicine-V for cough and sputum and is the main outcome measure in the Traditional Korean Medicine Clinical Practice Guidelines for Antitussives and Expectorants (Ministry of Food and Drug Safety, 2007). The questionnaire items were as follows: 1) cough frequency, intensity, and sensitivity; 2) sputum frequency, volume, difficulty coughing, appearance, and color; 3) activities of daily living; and 4) night-time sleeping. Each item is scored on a 4-point scale with a total maximum score of 40 points. The Leicester Cough Questionnaire (LCQ) evaluates the quality of life (QOL) associated with cough (Yousaf et al., 2011). It consists of 19 items divided into three parts: physical, psychological, and social; each scored from 1 to 7, and the higher the score, the better the QOL. We used the Leicester Cough Questionnaire-Korean version acute (LCQ-K-acute), which has proven its validity and reliability (Han et al., 2014). The QCSCS and LCQ were evaluated on Days 0 and 7, respectively. The Integrative Medicine Outcome Scale (IMOS) is a 5-point scale to evaluate improvement after treatment by the investigator, and the Integrative Medicine Patient Satisfaction Scale (IMPSS) is a 5-point scale to evaluate patient satisfaction after treatment by the patient. The lower the score, the better, and both were measured on Day 7.

### 2.7 Statistical Analysis

Data analysis was performed by an independent statistician using SAS Analytics Pro. The efficacy evaluation analysis in this study was mainly a full analysis set (FAS) analysis based on the intention-to-treat (ITT) analysis, and the per protocol (PP) analysis was the secondary analysis. For the primary outcome analysis, the changes in BSS between Days 0 and 7 were evaluated using analysis of covariance (ANCOVA), which contains the BSS of Day 0 and the treatment group as covariates. Analyses of secondary outcomes were carried out as follows: QCSCS and LCQ-K-acute by ANCOVA, including its baseline value, frequency of coughing fits by linear mixed models (LMMs), IMOS and IMPSS by analysis of variance (ANOVA), and withdrawal rate of patients with exacerbation by the Pearson’s χ2 or Fisher’s exact tests. To handle missing values when processing ITT analysis, the last-observation-carried-forward method was used except for the frequency of coughing fits analyzed with LMMs in which missing values will not be imputed. Safety assessment was performed using ITT analysis; all participants were randomized. Comparison of the number of AEs between the three treatment groups was performed using the Kruskal-Wallis test. Demographic and baseline data were analyzed using ANOVA for continuous variables and Pearson’s χ^2^ or Fisher’s exact tests for categorical variables. For the effects of GHX02 treatments compared with the control group, 95% CIs were calculated, and statistical significance was set at a two-sided test with an α-level of 0.05.

## 3 Results

### 3.1 Participants

**FIGURE 1:**
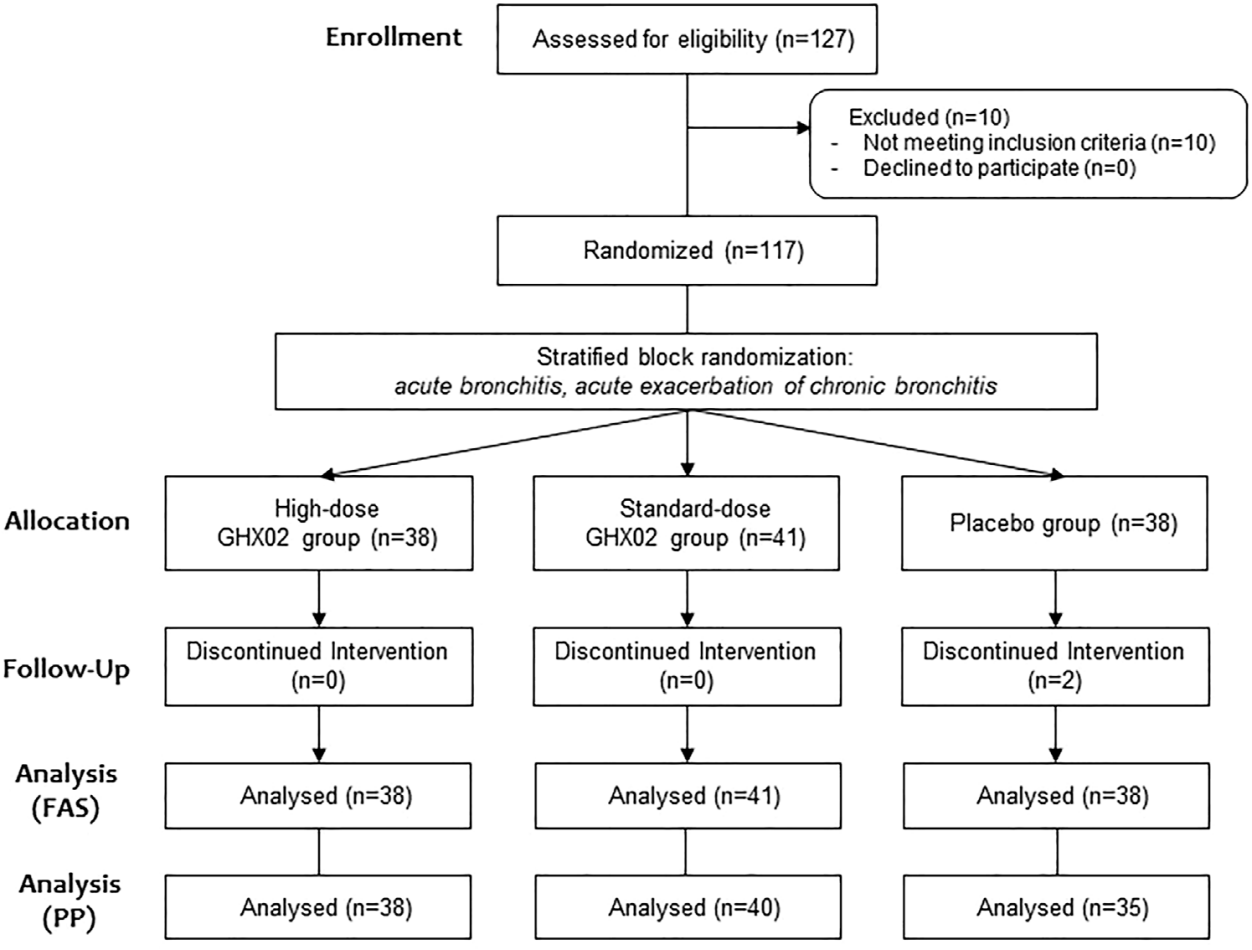
Flow chart of participants.

A total of 127 participants were screened in three hospitals from March 2019 to December 2019, and 10 participants were excluded from the study according to the exclusion criteria, and the remaining 117 were randomized to parallel groups. Of these, 38 subjects were placed in the high-dose GHX02 group, 41 subjects were placed in the standard-dose GHX02 group, and 38 subjects were placed in the placebo group. Two participants from the placebo group (one for error in urine test at the last visit, and one for exclusion criteria) dropped out after drug administration were included in FAS based on ITT analysis and excluded from PP analysis. Additionally, one participant from the control group and one from the high-dose GHX02 group were excluded from the PP analysis by the overdue visit period (Figure 1). There were no significant differences between the groups in the baseline demographics and clinical characteristics at baseline, except for weight, which was considered to have no clinical effects (Table 3).

**TABLE 3:** Baseline characteristics.

	GHX02 1920 mg/d (*n* = 38)	GHX02 960 mg/d (*n* = 41)	Placebo (*n* = 38)	*p*-value
Age (years)	38.4 ± 15.8	36.4 ± 13.1	38.6 ± 15.4	0.756^a^
Sex (N, male/female)	10/28	12/29	15/23	0.452^b^
Weight (kg)	59.8 ± 10.4	62.2 ± 9.2	69.6 ± 19.3	0.015^a^
Height (cm)	163.5 ± 7.5	164.7 ± 7.5	166.3 ± 9.3	0.311^a^
Smoker, current (N)	2	4	5	0.450^b^
Smoker, never (N)	36	37	32	
Smoker, former (N)	0	0	1	
Alcohol drinking (N)	12	11	12	0.905^b^
BSS	6.7 ± 1.7	6.4 ± 1.2	6.6 ± 1.4	0.511^a^

Values are expressed as mean ± SD.

a One-way ANOVA.

b Fisher’s exact tests.

### 3.2 BSS

**FIGURE 2:**
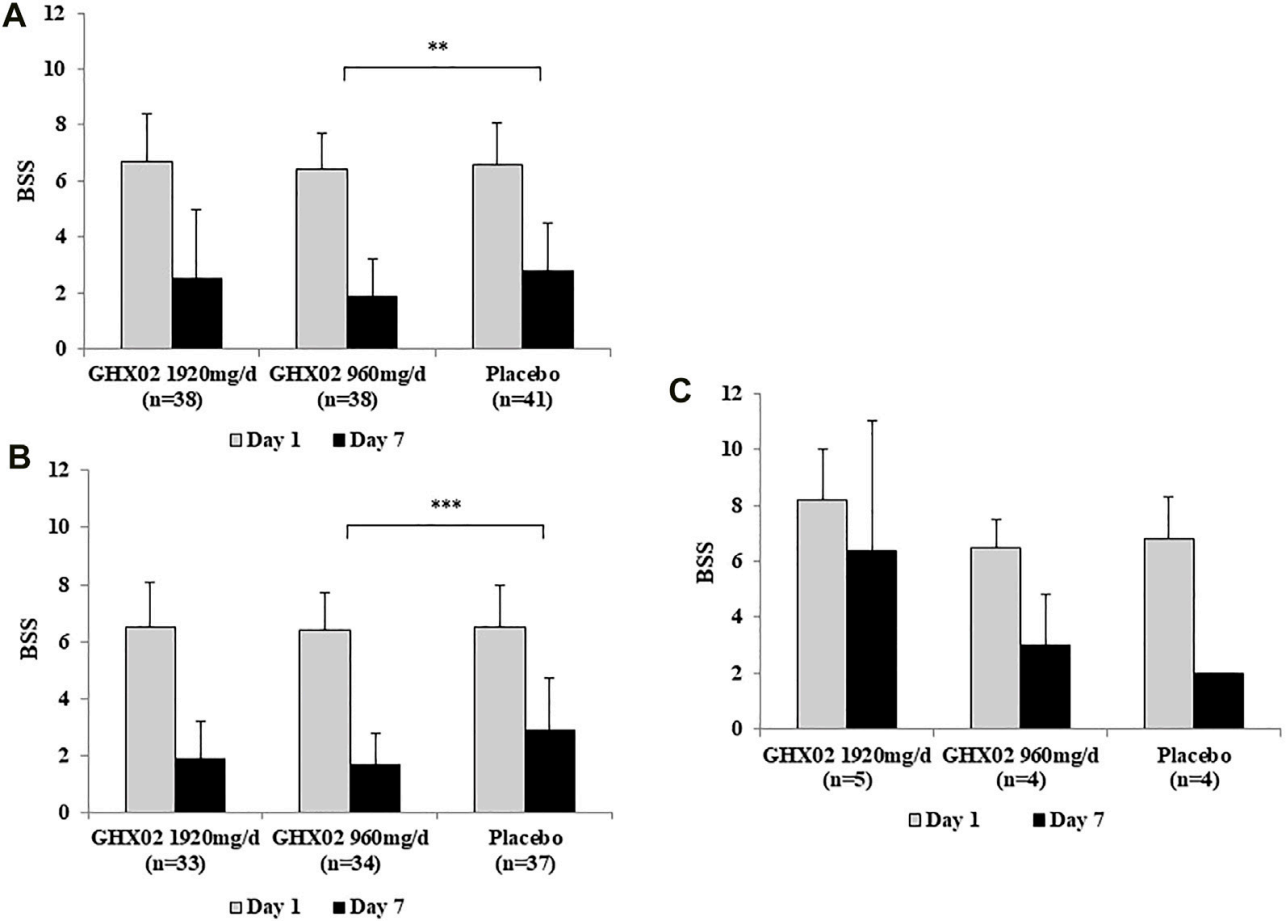
Figure 2. Bronchitis Severity Score (BSS) from Day 1 to Day 7. **(A)** Total population **(B)** AB subgroup **(C)** AECB subgroup. Results are expressed as mean ± SD with 95% confidence intervals (CIs) in FAS analysis. *p*-values indicate significance of mean differences in the value between groups (* *p* < 0.05, ** *p* < 0.01, and *** *p* < 0.001). AB, Acute Bronchitis; AECB, Acute Exacerbations of Chronic Bronchitis.

**FIGURE 3:**
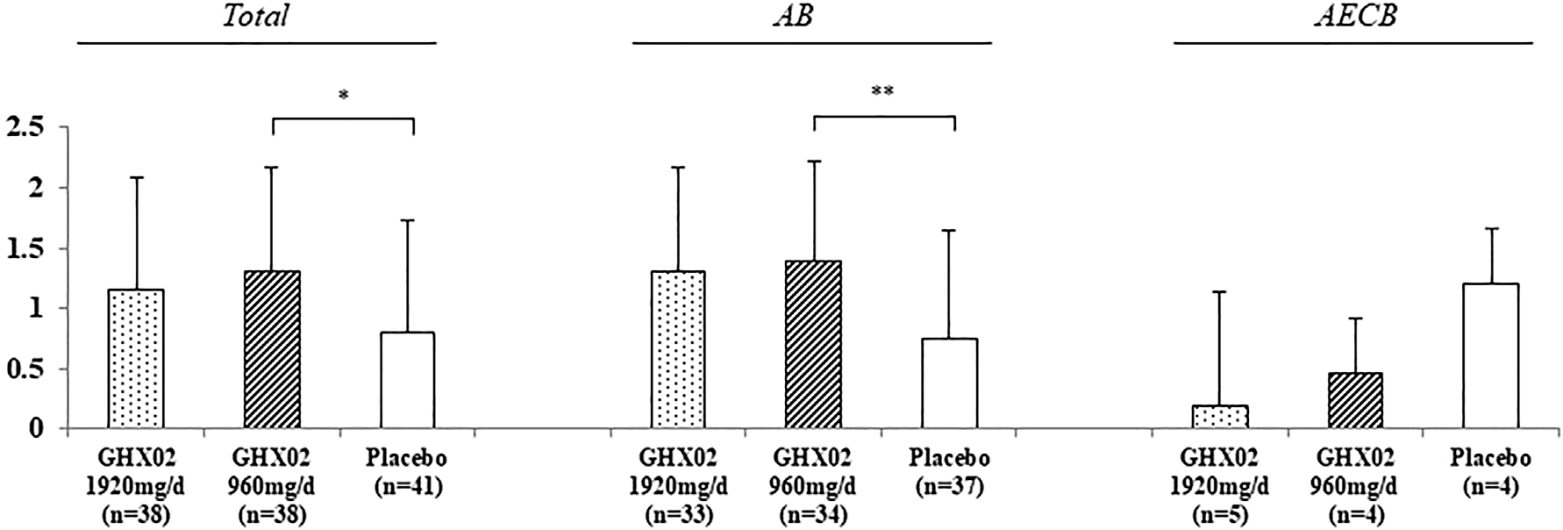
Change of Frequency of coughing fits from Day 1 to Day 7. Results are expressed as mean ± SD with 95% confidence intervals (CIs) in FAS analysis. *p*-values indicate significance of mean differences in the value between groups (* *p* < 0.05, ** *p* < 0.01, and *** *p* < 0.001). AB, Acute Bronchitis; AECB, Acute Exacerbations of Chronic Bronchitis.

The baseline BSS (visit 2) was 6.7 ± 1.7 in the high-dose GHX02 group, 6.4 ± 1.3 in the standard-dose GHX02 group, and 6.6 ± 1.5 in the placebo group. After 7-days treatment (visit 3), BSS was reduced to 2.5 ± 2.5 in the high-dose GHX02 group, 1.9 ± 1.3 in the standard-dose GHX02 group, and 2.8 ± 1.7 in the placebo group. The mean difference in the BSS from baseline to 7-days was 4.2 ± 2.0, 4.5 ± 1.8, and 3.8 ± 2.1 in the high-dose GHX02, standard-dose GHX02, and placebo groups, respectively (Figure 2A). The change in BSS between visits 2 and 3 was higher in the two treatment groups than in the placebo group. In particular, the mean difference was statistically significant between the standard-dose GHX02 and the placebo groups (95% CI, *p* = 0.028). In the subgroup analysis, the mean difference between visits 2 and 3 for acute bronchitis was statistically significant between the standard-dose GHX02 and placebo groups (95% CI, *p* = 0.0006) (Figure 2B). However, the mean difference between visits 2 and 3 for AECB was not statistically significant between the standard-dose GHX02 and placebo groups (95% CI, *p* = 0.553) (Figure 2C; Figure 3).

### 3.3 Frequency of Coughing Fits

**FIGURE 4:**
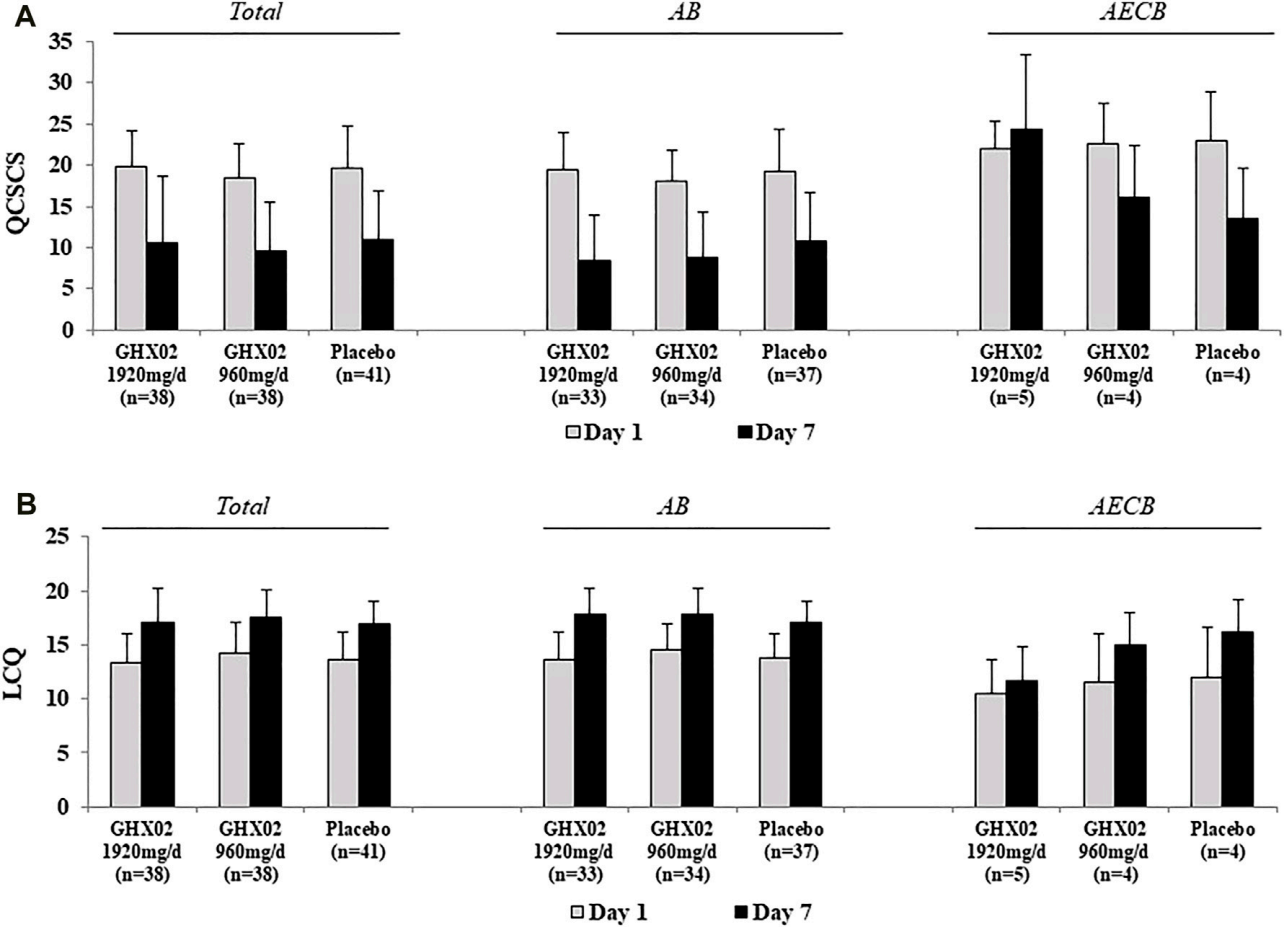
Questionnaire of Clinical Symptoms of Cough and Sputum (QCSCS) and Leicester Cough Questionnaire (LCQ) from Day 1 to Day 7. **(A)** QCSCS **(B)** LCQ. Results are expressed as mean ± SD with 95% confidence intervals (CIs) in FAS analysis. *p*-values indicate significance of mean differences in the value between groups (* *p* < 0.05, ** *p* < 0.01, and *** *p* < 0.001). AB, Acute Bronchitis; AECB, Acute Exacerbations of Chronic Bronchitis.

The frequency of coughing fits from baseline to Day 7 decreased from 4.3 ± 0.7 to 2.5 ± 1.5 in the high-dose GHX02 group, 4.0 ± 1.0 to 2.0 ± 1.1 in the standard-dose GHX02, and 3.9 ± 0.8 to 2.3 ± 1.4 in the placebo group. The frequency of coughing fits gradually decreased over 7 days, and the mean differences from baseline to Day 7 were statistically significant between the standard-dose GHX02 and placebo groups in the total population (95% CI, *p* = 0.036) and Acute bronchitis (AB) subgroup analysis (95% CI, *p* = 0.0045) (Figure 4). However, the mean differences between the groups in the AECB subgroup analysis were not statistically significant.

### 3.4 QCSCS

The QCSCS from baseline to 7-days treatment decreased from 19.8 ± 4.4 to 10.5 ± 8.1, 18.5 ± 4.0 to 9.6 ± 5.9, and 19.7 ± 5.1 to 11.0 ± 5.9 in the high-dose GHX02, standard-dose GHX02, and placebo groups, respectively. The mean difference in the high-dose GHX02 group, standard-dose GHX02, and placebo groups (Figure 4A) was 9.4 ± 7.5, 8.9 ± 5.7, and 8.9 ± 6, respectively. However, there was no statistically significant difference, and the subgroup analysis did not show any significant differences.

### 3.5 LCQ

At baseline, LCQ was 13.3 ± 2.8 in the high-dose GHX02 group, 14.3 ± 2.8 in the standard-dose GHX02 group, and 13.6 ± 2.6 in the placebo group. After 7-days treatment, LCQ increased by 17.1 ± 3.2, 17.6 ± 2.5, and 17.0 ± 2.1 in the high-dose GHX02, standard-dose GHX02, and placebo groups, respectively. The mean difference was 3.9 ± 3.2 in the high-dose GHX02 group, 3.3 ± 2.3 in the standard-dose GHX02 group, and 3.4 ± 2.8 in the placebo group (Figure 4B). There was an increase in QOL associated with cough in all groups, but there were no statistically significant differences between the groups, which was the same in the subgroup analysis.

### 3.6 IMOS and IMPSS

**FIGURE 5:**
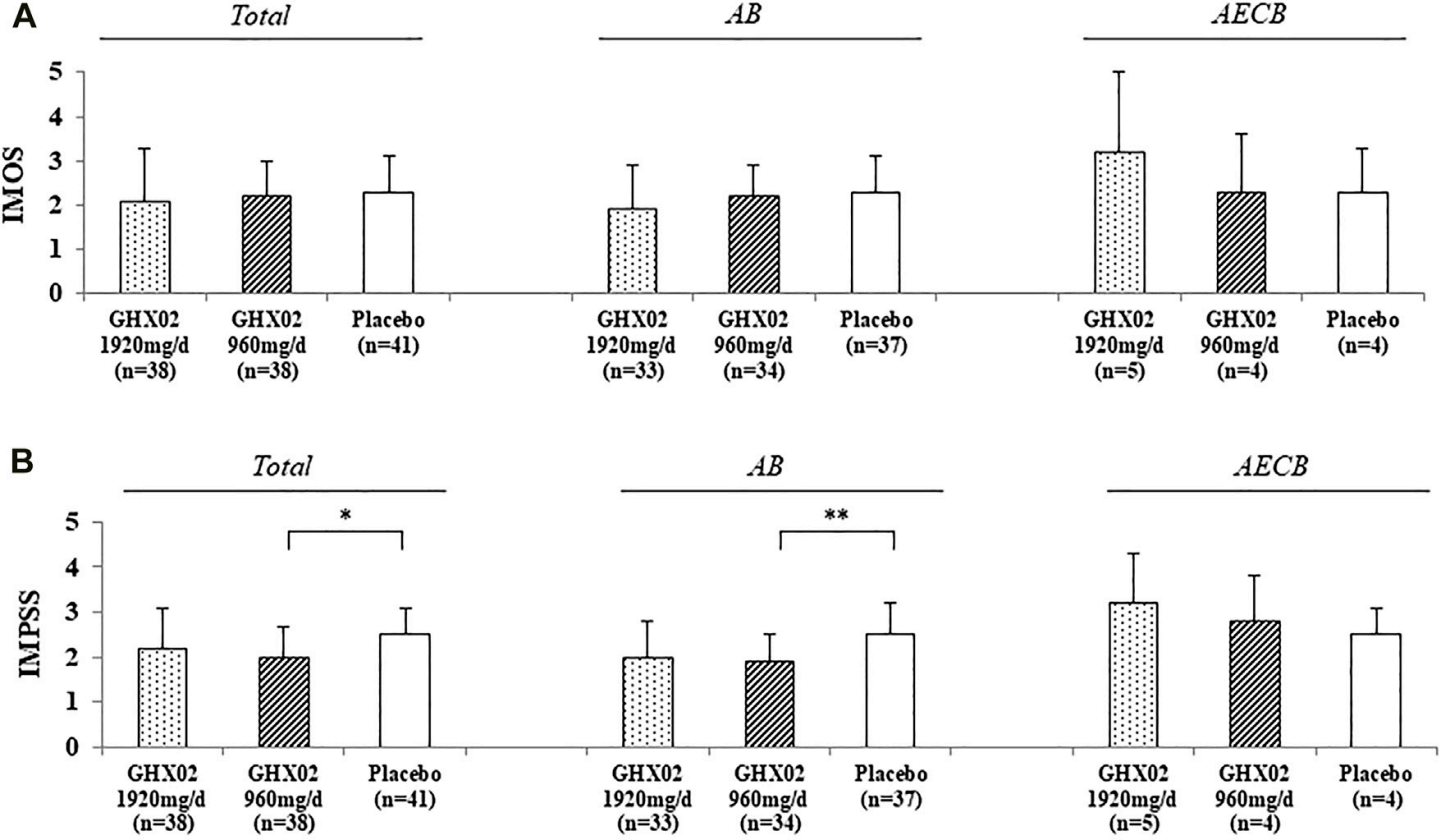
Integrative Medicine Outcome Scale (IMOS) and Integrative Medicine Patient Satisfaction Scale (IMPSS) in Day 7. **(A)** IMOS **(B)** IMPSS. Results are expressed as mean ± SD with 95% confidence intervals (CIs) in FAS analysis. *p*-values indicate significance of mean differences in the value between groups (* *p* < 0.05, ** *p* < 0.01, and *** *p* < 0.001). AB, Acute Bronchitis; AECB, Acute Exacerbations of Chronic Bronchitis.

The IMOS and IMPSS scores were evaluated after 7-days treatment (Figure 5). The IMOS on Day 7 was 2.1 ± 1.2, 2.2 ± 0.8, and 2.3 ± 0.8 in the high-dose GHX02, standard-dose GHX02, and placebo groups, respectively. There were no statistically significant differences between the groups (95% CI, *p* = 0.625). The IMPSS on Day 7 was 2.2 ± 0.9, 2.0 ± 0.7, and 2.5 ± 0.6 in the high-dose GHX02, standard-dose GHX02, and placebo groups groups, respectively. There was a statistically significant difference between the standard-dose GHX02 and the placebo groups (95% CI, *p* = 0.027), which means that the patients in the standard-dose GHX02 group were more satisfied than those in the placebo group (Figure 5A). In the subgroup analysis, IMPSS for the AB subgroup was statistically significant between the standard-dose GHX02 and placebo groups (95% CI, *p* = 0.0027), and IMOS for the AB subgroup, IMOS, and IMPSS for the AECB group were not statistically significant between the three groups (Figures 5B,C).

### 3.7 AEs

In the FAS, 12 of the 117 participants experienced AEs during the trial. There were four cases in the high-dose GHX02 group, three cases in the standard-dose GHX02 group, and five cases in the placebo group; these differences were not statistically significant (*p* = 0.694). Twelve AEs were considered mild and there were no severe AEs. The most common AE was gastrointestinal disorder (diarrhea or dyspepsia), and all other AEs were confirmed to be fully resolved after the trial, except for one minor increase in aspartate aminotransferase (AST) and alanine aminotransferase (ALT) that failed to follow-up. None of the AEs required study drug discontinuation or dropout.

## 4 Discussion

Both acute bronchitis and AECB have cough and sputum as the main symptoms, and the prevalence is high enough to be common in primary care and is also increasing due to air pollution such as particulate matter (Kampa and Castanas, 2008). Consequently, the demand for antitussives and expectorants increases, but the evidence on the appropriateness of antibiotic prescriptions and the effect of symptomatic treatment for bronchitis is unclear. In particular, the excessive use of antibiotics for acute bronchitis, which is mostly caused by viruses, only leads to increased resistance strains of common organisms, adverse effects on normal bacteria colonizing the intestine, such as gastrointestinal symptoms, and medical costs (Smith et al., 2017). Antitussives and expectorants for symptomatic treatment also have adverse effects on the central nervous system, such as respiratory depression, nausea, dizziness, and uncertain effectiveness (Bolser, 2006). Therefore, we conducted this clinical trial to establish the basis for an effective bronchitis treatment using herbal medicines with fewer adverse effects that contribute to the reduction of the medical cost burden.

GHX02, which contains four herbs, is a combined formulation that has been clinically used for bronchitis at Daejeon Korean Medicine Hospital, and Daejeon University. In preclinical studies, GHX02 decreased the frequency of coughing and exhibited expectorant activity and antimicrobial activity against *Streptococcus pneumoniae* in a mouse model. GHX02 also suppressed histamine release from mast cells and reduced leukocyte levels, prostaglandin E2 (PGE2), interleukin (IL)-4, and IL-13. Furthermore, GHX02 suppressed PM_10_D-induced inflammatory symptoms in the lungs, such as increased alveolar wall thickness, cytokine release, and collagen fiber accumulation (Yang et al., 2020). In addition, we observed that GHX02 efficiently inhibited airway inflammation by inhibiting the expression of proinflammatory cytokines and the migration of inflammatory cells in a COPD-induced mouse model (Yang et al., 2018). In a subsequent study investigating the mechanism, the GHX02 herbal formula protects against TNF-α-induced inflammation in human bronchial epithelial cells by blocking NF-κB and activating the Nrf-2/HO-1 pathway.

In this study, which is the first human clinical trial of GHX02, we evaluated the efficacy and safety of GHX02 for the treatment of acute bronchitis and AECB in a randomized, double-blind, dose-finding phase II, placebo-controlled, multicenter trial. The primary outcome measure in this study was the BSS, which was evaluated based on the patient’s subjective symptoms. Two different doses of GHX02 (1920 mg/day, 960 mg/day) were effective in decreasing the BSS from baseline to 7-days treatment compared to placebo. The mean difference in the BSS was 4.2 ± 2.0 in the high-dose GHX02 group, 4.5 ± 1.8 in the standard-dose GHX02 group, and 3.8 ± 2.1 in the placebo group (Figure 2). These results are consistent with those of previous studies that used herbal medicines (Kamin et al., 2010; Park et al., 2017). Considering that the BSS is used to diagnose acute bronchitis with 5 points more and is a valid clinical measure for acute bronchitis treatment (Matthys and Kamin, 2013), the results of this study can be judged as a clinically significant improvement. In secondary outcome measures, IMPSS, which evaluates patient satisfaction after treatment, and showed a statistically significant difference between the groups. The IMPSS on Day 7 was 2.0 ± 0.7 in the standard-dose GHX02 group and 2.5 ± 0.6 in the placebo group. These results were in line with those of other clinical trials that used BSS as the primary outcome measure and IMPSS as the secondary outcome measure (Kardos et al., 2014). As the BSS has subjective components, there may be individual variations. However, the BSS is supported by the results of additional outcome measures, such as IMPSS, which focus on general outcomes and patient satisfaction with the treatment (Matthys and Kamin, 2013). In addition, the frequency of coughing fits gradually decreased over 7 days in all groups, and the mean differences from baseline to Day 7 were statistically significant between the standard-dose GHX02 and the placebo groups. The frequency of coughing fits is an objective indicator of the main symptoms of bronchitis, and its change means that the treatment group showed improved clinical symptoms compared to the placebo group.

In the other outcome measures, there were no statistically significant differences between the groups, but the high-dose GHX02, and standard-dose GHX02 groups showed more clinical improvement than the placebo group. The QCSCS that evaluates cough, sputum, daily living, and sleeping was not reported for its minimum clinically important difference (MCID) but decreased by half in the order of high-dose GHX02, standard-dose GHX02, and placebo groups. In the LCQ, which is the most frequently used questionnaire for QOL related to cough, the MCID was reported as 2–2.5 points (Lee et al., 2013; Kardos et al., 2014). Therefore, the results of this study are clinically significant in that the LCQ showed a change of 3.9 ± 3.2 points in the high-dose GHX02 group and 3.3 ± 2.3 in the standard-dose GHX02 group. The IMOS was better in the order of high-dose GHX02, standard-dose GHX02, and placebo groups. Since cough affects not only the physical aspect but also the psychological and social life, it is necessary to comprehensively evaluate all parts to measure the degree of cough (Brignall et al., 2008). In this study, we evaluated both objective and subjective aspects of symptoms using several outcome measures. The BSS, which is based on the investigator’s objective clinical evaluation in combination with the subjective feedback of the patient, showed significant improvement as the primary outcome measure. Although there were clinical improvements, IMPSS and the frequency of coughing fits in objective or subjective secondary outcome measures did not show statistically significant differences between the groups. These effects in the placebo group may be due to the placebo effect, and to evaluate the efficacy of GHX02 more accurately, further trials should be conducted.

In terms of safety, there were no serious AEs in this study, and several mild AEs were not clinically significant. The vital signs and laboratory examinations assessed before and after taking the GHX02 did not change significantly between the groups. Both preclinical and clinical trials have indicated that GHX02 is safe during the drug-taking period.

This study has several limitations. First, we stratified block randomization into acute bronchitis and AECB, but in the AECB subgroup, and the number of subjects was not enough to compare the differences between the groups. In this trial, the number of patients with acute bronchitis was 104, while the total number of patients with AECB was 13. Since only the efficacy for acute bronchitis was confirmed, and for AECB was not confirmed, future studies targeting AECB may be independently conducted. Second, only adults were involved in this study; children were excluded because clinical drugs are recommended to be first applied to adults. However, the prevalence of bronchitis is high in children, and the adverse effects of central antitussives, such as codeine, are more severe in children (Malesker et al., 2017). Therefore, further trials targeting children need to be conducted. Third, BSS is used to diagnose and evaluate the impact of treatment in clinical trials for acute bronchitis, but it would be beneficial if a biomarker was developed additionally. BSS and other questionnaires can also evaluate acute bronchitis as a validation tool, but developing biomarkers will provide more objective evidence.

Despite these limitations, this is a meaningful study that raises the possibility of generalization by creating scientific grounds for Korean medicine used in clinical practice. By comparing the two different doses of GHX02, the most effective dose was considered 960 mg/day. In the next phase III study, it will be necessary to specify the target disease as acute bronchitis, which has been proven effective in this trial, and compare it with the active control group for sufficient evidence.

## 5 Conclusion

In conclusion, this study suggests that GHX02 has clinical improvements in patients with acute bronchitis and can be used as a safe and effective remedy for acute bronchitis. Our data will be used as clinical evidence to plan a phase III confirmatory clinical trial to evaluate the efficacy of GHX02 for acute bronchitis compared with an active control group.

## Data Availability

The raw data supporting the conclusion of this article will be made available by the authors, without undue reservation.
